# A Review of Molecular Interplay between Neurotrophins and miRNAs in Neuropsychological Disorders

**DOI:** 10.1007/s12035-022-02966-5

**Published:** 2022-08-02

**Authors:** Sara Abdolahi, Ameneh Zare-Chahoki, Farshid Noorbakhsh, Ali Gorji

**Affiliations:** 1 Shefa Neuroscience Research Center, Khatam Alanbia Hospital, Tehran, Iran; 2 Neuroscience Research Center, Institute of Neuropharmacology, Kerman University of Medical Sciences, Kerman, Iran; 3 Department of Immunology, School of Medicine, Tehran University of Medical Sciences, Tehran, Iran; 4 Neuroscience Research Center, Mashhad University of Medical Sciences, Mashhad, Iran; 5 Department of Neurosurgery, Westfälische Wilhelms-Universität, Münster, Germany; 6 Department of Neurology and Institute for Translational Neurology, Westfälische Wilhelms-Universität, Münster, Germany; 7 Epilepsy Research Center, Westfälische Wilhelms-Universität, 48149 Münster, Germany

**Keywords:** Brain, Neurotrophic Factors, CNS, Psychological Disorders, NT3, BDNF

## Abstract

Various neurotrophins (NTs), including nerve growth factor, brain-derived neurotrophic factor, neurotrophin-3, and neurotrophin-4, promote cellular differentiation, survival, and maintenance, as well as synaptic plasticity, in the peripheral and central nervous system. The function of microRNAs (miRNAs) and other small non-coding RNAs, as regulators of gene expression, is pivotal for the appropriate control of cell growth and differentiation. There are positive and negative loops between NTs and miRNAs, which exert modulatory effects on different signaling pathways. The interplay between NTs and miRNAs plays a crucial role in the regulation of several physiological and pathological brain procedures. Emerging evidence suggests the diagnostic and therapeutic roles of the interactions between NTs and miRNAs in several neuropsychological disorders, including epilepsy, multiple sclerosis, Alzheimer’s disease, Huntington’s disease, amyotrophic lateral sclerosis, schizophrenia, anxiety disorders, depression, post-traumatic stress disorder, bipolar disorder, and drug abuse. Here, we review current data regarding the regulatory interactions between NTs and miRNAs in neuropsychological disorders, for which novel diagnostic and/or therapeutic strategies are emerging. Targeting NTs-miRNAs interactions for diagnostic or therapeutic approaches needs to be validated by future clinical studies.

## Introduction

The mammalian neurotrophins (NTs), a family of structurally-related proteins, regulate neurite outgrowth and modulate neuronal differentiation and survival [1]. The NT family consists of four proteins: brain-derived neurotrophic factor (BDNF), nerve growth factor (NGF), neurotrophin-3 (NT3), and neurotrophin-4 (NT4), which are important in the regulation of various physiological and pathological conditions [2]. There are two different classes of receptors that are activated by NTs, *(i)* the p75 neurotrophin receptors (p75NTR), which are a low-affinity NTs receptors, and *(ii)* the tropomyosin-related kinase receptors (Trk), which are high-affinity NTs receptors and consist of TrkA, TrkB, and TrkC [3, 4]. NGF has the highest affinity to TrkA, BDNF and NT4 stimulate preferentially TrkB, and NT3 acts mainly through TrkC. Several diverse intracellular signaling pathways may be affected by the activation of Trk, including the phosphatidylinositol 3-kinase-protein kinase B (PI3K-Akt) signaling pathway, extracellular-signal-regulated kinase 1/2 (ERK1/2), PI3K, phospholipase C, and mitogen-activated protein kinase (Ras/Raf/MAPK) pathway [5–8]. The p75NTR is a multifunctional transmembrane protein, which can unselectively bind to all mature NTs [9]. The p75NTR couple to different intracellular binding proteins, activate signaling adaptors, and modulate the Trk signaling pathway. The p75NTR contributes to the neurogenesis of adult neural progenitors via NGF activation [10, 11]. Several signaling elements of the Trk pathways mediate NT functions, particularly gene transcription regulation [3]. Moreover, NTs are implicated in the regulation of several transcription factors (TFs), like cAMP response element-binding protein B (CREB) and nuclear factor kappa B (NF-κB) [12]. There are functional connections between NTs, TFs, and their transcriptional targets [13]. Enhancement of NT expression in the brain could protect neuronal tissues against a variety of pathological insults, such as ischemic and traumatic events as well as neurodegenerative processes [14, 15]. NTs and their receptors are also involved in neuropsychiatric disorders [16].

The bidirectional interactions between NTs and brain-specific microRNAs (miRNAs) regulate the expression of numerous protein-encoding genes [17]. miRNAs, a class of tiny non-coding RNAs with a length of approximately 22 nucleotides, are the major post-transcriptional regulators of gene expression [18]. miRNA precursors are located within both intragenic and intergenic regions of DNA. miRNAs function involves a multi-step process, including transcription and processing of primary miRNAs, precursor-miRNAs hairpin formation, and export of mature miRNAs from the cytoplasm to the nucleus [19, 20]. Multiple factors play a regulatory role in these processes, including RNA polymerase II, rosha, Exportin 5, Dicer, and Argonaute [21]. Mature miRNA can be sorted and loaded into the Argonaute proteins to form an RNA-induced silencing complex [22].

Approximately 30–60% of all mammalian proteins can be targeted by miRNAs, which are implicated in various cellular and developmental processes. miRNAs regulate cell proliferation, differentiation, regeneration, and cell death [23, 24]. miRNAs and their abundant targets also play a pivotal role in neural lineage and subtype determination as well as neural stem cell development in both physiological and pathological states [25]. Circulating miRNAs are released into the extracellular fluids, such as blood, urine, and cerebrospinal fluid (CSF) [26]. Growing evidence suggests that brain-specific miRNAs play an important role in the regulation of neuronal activity [27]. Furthermore, miRNAs mediate neuronal communication via regulating the protein synthesis that is implicated in synaptic transmission [28]. miRNA dysregulation in the nervous system could affect a wide range of biological functions, such as neurogenesis, myelination, and dendritic outgrowth [29, 30]. Dysregulation in the miRNA signaling disrupts the functions of neurons [31], astrocytes [32], microglia [33], oligodendrocytes [30], and ependymal cells [34]. miRNAs are implicated as biologically crucial mediators in the pathogenesis of various neuropsychological diseases, such as Alzheimer’s disease (AD), Huntington’s disease (HD), Parkinson’s disease (PD), epilepsy, anxiety, depression, schizophrenia, post-traumatic stress disorder (PTSD), bipolar disorder, and substance abuse [31, 35–37]. miRNAs could serve as a potential biomarker for the early detection of various neurodegenerative disorders [26].

Both NTs and brain-specific miRNAs play a potential role in diagnostic and therapeutic approaches for central nervous system (CNS) disorders. Indeed, a strong relationship between the regulation of NTs-miRNAs and the pathophysiology of various brain disorders has been determined [38–40]. Here, we summarize the current understanding of the regulatory mechanisms of NTs and miRNAs interactions. Furthermore, we provide a comprehensive review of the current knowledge regarding the potential diagnostic, predictive, prognostic, and/or therapeutic roles of the NTs and miRNAs interactions in neuropsychological disorders.

## Molecular Interactions between NTs and miRNA Signaling

miRNAs are considered one of the important regulators in eukaryotic transcription [41]. Various brain-specific miRNAs are known to play a critical role in NT expression and function [31]. NTs and miRNAs mutually regulate each other. NT expression is not only regulated by miRNAs, but it, in turn, modulates miRNA expression [42, 43]. NTs are involved in a wide range of gene expressions mostly at the level of transcription and translation [44]. NTs modulate MAPK/ERK pathways and control miRNA levels. The modulation of MAPK/ERK could alter miRNA values through (i) phosphorylation of TAR RNA binding protein and Dicer, (ii) regulation of CREB and NF-κB, and (iii) alteration of Lin-28 homolog A [17]. A given miRNA may directly regulate multiple mRNAs, each of them has different binding sites to promote the binding performance [45].

### Effect of miRNAs on NT Expression

Brain-enriched miRNAs have been described to play a critical role in NT expression. NTs regulate neuronal and synaptic functions during development and adulthood, and miRNAs modulate NTs [46–48]. miRNAs target different mRNAs through the interaction with the 3′ untranslated regions (3′ UTR); however, binding to other regulatory regions of mRNA can also occur [43, 49]. The direct or indirect interactions between miRNAs and their target genes can be influenced by multiple factors [50]. Some miRNAs mediate NTs expression through post-transcriptionally regulating TFs expression. CREB, as a transcription factor (TF), stimulates transcription in association with CREB-binding protein and its homolog p300. CREB could also bind to various BDNF promoter elements and enhance the NT activities [17, 41, 51]. For instance, miR-134 directly targets CREB mRNA and inhibits its translation. Inhibition of CREB signaling could abolish the BDNF expression [17].

miRNAs modulate NTs in an isoform-specific manner. An in vitro study on the human neuroblastoma cell line SH-SY5Y has shown that an isoform of the neurotrophic receptor tyrosine kinase 3 (NTR3) is specifically regulated by different sets of miRNAs. Overexpression of miR-128 regulates the truncated isoform of NTR3 (t-NTR3) and miR-151-3p regulates the full-length isoform of NTR3 at the mRNA level [46]. In vitro analysis of TrkC expression in retinoic acid (RA)-treated SK-N-BE cells indicated that t-NTR3 mRNA can be targeted by miR-9, miR-125a, and miR-125b. A regulatory circuitry involving these miRNAs and TrkC has been identified to play a key role in controlling cell proliferation [52]. miRNAs can also regulate NTs receptors under certain conditions. Upregulation of P75NTR is implicated in the pathogenesis of brain injury and apoptosis. miR-592 could regulate p75NTR at the mRNA level, and an inverse relationship is defined between miR-592 and p75NTR [17, 53]. We have identified various miRNAs that can directly target NTs in humans and mice using the miRTarBase database (Table 1).

**Table 1:** miRNAs-neurotrophins interactions evaluated by miRTarBase database. *BDNF*, brain-derived neurotrophic factor; *NGF*, nerve growth factor; *NT 3*, neurotrophin-3; *NT 4*, neurotrophin-4; *NA*, not available

Target	Species (target)	Species (miRNA)	miRNA	ID	Validation methods
BDNF	*Homo sapiens*	*Homo sapiens*	hsa-miR-124-3p	MIRT000362	Reporter assay
	*Homo sapiens*	*Homo sapiens*	hsa-miR-30a-5p	MIRT001946	Reporter assay
	*Homo sapiens*	*Homo sapiens*	hsa-miR-1-3p	MIRT002955	Reporter assay
	*Homo sapiens*	*Homo sapiens*	hsa-miR-210-3p	MIRT003153	Reporter assay
	*Homo sapiens*	*Homo sapiens*	hsa-miR-22-3p	MIRT005900	Reporter assay
	*Homo sapiens*	*Homo sapiens*	hsa-miR-204-5p	MIRT437447	Reporter assay
	*Homo sapiens*	*Homo sapiens*	hsa-miR-16-5p	MIRT437463	Reporter assay
	*Homo sapiens*	*Homo sapiens*	hsa-miR-1-5p	MIRT732282	Reporter assay
	*Mus musculus*	*Mus musculus*	mmu-miR-381-3p	MIRT004768	Reporter assay
	*Mus musculus*	*Mus musculus*	mmu-miR-495-3p	MIRT004769	Reporter assay
	*Mus musculus*	*Mus musculus*	mmu-miR-30a-5p	MIRT004770	Reporter assay
	*Mus musculus*	*Mus musculus*	mmu-miR-30d-5p	MIRT004771	Reporter assay
	*Mus musculus*	*Mus musculus*	mmu-miR-206-3p	MIRT005406	Reporter assay
NGF	*Homo sapiens*	*Homo sapiens*	-	-	NA
	*Mus musculus*	*Mus musculus*	-	-	NA
NT3	*Homo sapiens*	*Homo sapiens*	-	-	NA
	*Mus musculus*	*Mus musculus*	-	-	NA
NT4	*Homo sapiens*	*Homo sapiens*	-	-	NA
	*Mus musculus*	*Mus musculus*	-		NA

#### miRNAs Interactions with NGF, BDNF, and NT3

miRNAs can modulate the expression of NGF, BDNF, and NT3. Multiple studies have revealed the role of miRNA downstream on NGF to regulate cell proliferation and/or apoptosis and consequently modulate neuronal differentiation [54]. miR-200 inhibits cell proliferation and promotes cell differentiation and neurite formation via targeting TF SRY-box transcription factor 2 and kruppel-like factor 4 [55]. Studies on developing rat brains as well as pheochromocytoma cell lines (PC12) provide supporting evidence that miR-29a and miR-29c increase neurite outgrowth through direct inhibition of tumor suppressor gene phosphatase and tensin homolog expression [56]. In PC12 cells, miR-200 targets some TFs and induces a neural marker, neurofilament light polypeptide [55, 57]. Previous studies have also demonstrated that miR-183 and/or miR-96 inhibit NGF-treated PC12 differentiation [58]. Furthermore, miR-221 plays a pivotal role in the NGF signaling, and overexpression of miR-221 can replace NGF in neural differentiation and survival [59]. miR-21 maintains the NGF effect on neuronal survival [60].

miR-155 upregulation enhances NGF expression at the protein level and its downregulation inhibits cytokine signaling 1 expression and NF-κB activation (Table 2) [61]. Knockout of miR-204/211 increases NGF expression at the mRNA level and activates the Akt signaling pathway (Table 2) [62]. miR-455-3p can also directly target NGF mRNA [63]. Another study suggests that lethal (Let)-7-5p is an upstream regulator of NGF [64]. In vivo studies elucidate that let-7 and miR-675 can directly target P53 and NGF mRNA [65–67]. Transfection of human dorsal root ganglia cell culture with miR-455-3p significantly reduced NGF expression at the mRNA level, which was reversible after the application of a miR-455-3p inhibitor (Table 2) [68]. Moreover, downregulation of miR-125b reversely increases NGF expression at the mRNA and protein levels (Table 2) [69]. Chronic inflammatory pain leads to the upregulated expression of miR-29b, which promotes the demethylation at the promoter region of the NGF gene, resulting in the upregulation of NGF gene expression (Table 2) [70]. Analysis of CSF of patients infected with acute viral encephalitis has shown overexpression of miR-150-5p that negatively correlated with transforming growth factor-β, NGF, axon guidance, and MAPK [71]. An experimental study on an acute cerebral ischemia model indicates that upregulation of miR-381 inhibits leucine-rich repeat containing-4 via the stromal cell-derived factor-1/C-X-C chemokine receptor type 4 signaling, enhances NGF protein expression, prevents neuronal apoptosis (Table 2) [72].

**Table 2:**
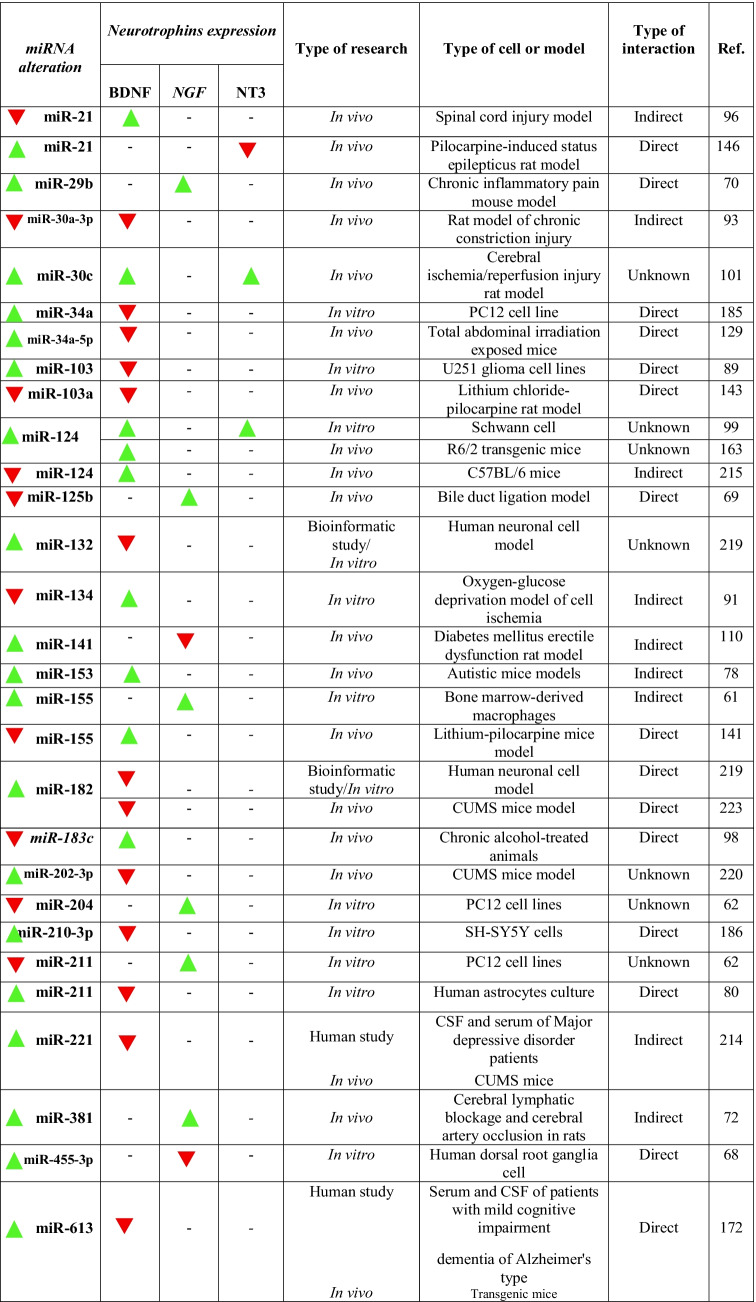
Effects of microRNAs on neurotrophins expression

*CUMS*, chronic unpredictable mild stress. Green triangle, upregulation; red inverted triangle, downregulation

Both miRNAs and BDNF play a role in the regulation of brain synaptic plasticity. Deletion of various brain-specific miRNAs, such as miR-124, miR-132, miR-137, miR-138, miR29a, and miR29c, increased hippocampal synaptic transmission as well as the expression of BDNF protein. Different miRNAs, such as miR-15a, miR-206, and miR-210, directly regulate BDNF protein expression and activity [12, 73]. Moreover, several investigations revealed that BDNF mRNA is the direct target of miR-10b, miR-19, miR-22, miR-26a-1, miR-26a-2, miR-26b, miR-195, and miR-30a-5p [74–76]. miR-15a has been suggested to inhibit the proliferation of neuronal cells and promote cell apoptosis by targeting BDNF mRNA and protein through downregulation of the PI3K/AKT pathway [77]. The high expression of miR-153 by targeting leptin receptors significantly suppresses the Janus kinase/signal transducers and activators of the transcription signaling pathway and thereby enhances BDNF expression at the mRNA and protein levels and neuronal proliferation (Table 2) [78]. Overexpression of miR-10a by targeting the BDNF signaling pathway could inhibit cell proliferation and induce neuronal apoptosis in the hippocampus [79]. Upregulation of miR-211 significantly inhibits BDNF mRNA and protein expression and suppresses the viability and proliferation of normal human astrocytes via the activation of lipopolysaccharides and the PI3K/Akt pathway (Table 2) [80]. Methyl CpG binding protein 2 (MeCP2) is one of the important genes in the maturation of new neurons and its dysregulation plays a role in the pathophysiology of Rett syndrome [81].

MeCP2 is a transcriptional regulator of BDNF [82]. The BDNF protein level was reduced in the MeCP2 mutant mice and an increase in BDNF levels improved motor skills in both mutant mice and children with Rett syndrome [83]. It was demonstrated that deletion of BDNF in MeCP2 mutations caused an earlier onset of overt symptoms in patients with Rett syndrome [84]. Several investigations provide evidence regarding the functional interaction between MeCP2 and BDNF. The modulation of BDNF pathways has been suggested as a potential strategy for treating children with Rett syndrome [85]. MeCP2 also regulates miR-15a and its reduction leads to abnormality in dendrite morphology during neurogenesis. On the other hand, miR-15a regulates BDNF expression at the mRNA and protein levels and exogenous BDNF can partially compensate for miR-15a deficiency during neuron maturation [86].

BDNF and miR-124 play a critical role in the pathogenesis of acute ischemic stroke. Contrary to other investigations, a negative correlation has been observed between serum BDNF and miR-124 values in patients with ischemic stroke [87]. Several miRNAs might act as diagnostic, prognostic, and/or therapeutic biomarkers for human gliomas [88]. A negative regulatory correlation between miR-103 and the BDNF mRNA and protein expression levels has been reported in gliomas. Overexpression of miR-103 inhibits the proliferation and invasion of cancer cells in patients with gliomas through downregulation of BDNF (Table 2) [89]. The oxygen–glucose deprivation/reoxygenation enhances miR-1 expression, which directly suppresses the expression of BDNF mRNA and protein, and subsequently affects cell survival and apoptosis [90]. Downregulation of miR-134 reduces ischemic injury through upregulation of CREB and downstream genes, including BDNF and Bcl-2, in ischemic hippocampal neurons (Table 2) [91].

Experimental evidence indicates that alterations of miRNAs contribute to neuropathic pain [92]. Using in vitro model of chronic constriction injury, it has been shown that decreased miR-30a-3p contributes to neuropathic pain. This study suggested that miR-30a-3p may inhibit BDNF activation via targeting the acetylated histone H3 and H4 on its promoter (Table 2) [93]. Investigations on animal and human embryonic stem cell (hESC)-derived neurons have revealed the association between anesthesia-induced neural injury and increasing hsa-miR-375 and miR-170 levels. These studies indicated that the BDNF gene is directly and reversely regulated by hsa-miR-375 and miR-170 and its upregulation protects neurons from anesthesia-induced neuronal cell damage and neural toxicity [94, 95]. The reduction of miR-21 level in a mice model of the spinal cord injury led to the upregulation of BDNF gene expression (Table 2) [96].

Resveratrol, a potent silent information regulator 1 (Sirt1), downregulates miR-134 and consequently causes an increase in CREB/BDNF expression levels in the hippocampus and improves hippocampal-dependent learning and memory [97]. In chronic alcohol-treated animals, downregulation of miR-183c in association with overexpression of BDNF mRNA exhibits a neuroprotective effect (Table 2) [98]. Overexpression of miR-124 in Schwann cells significantly enhances the BDNF and NT3 mRNA expression, which might involve in neuron development processes (Table 2) [99]. NT3 and BDNF are predictive targets for miR-182 upregulation that might negatively control NT3 and BDNF expression in ancestral stress-induced behaviors [100]. miR-30c transfection can also improve neuronal injury and increase NT3 and BDNF expression in the rat hippocampus (Table 2) [101]. miR-200c and miR-429 also directly target NT3 mRNA [102, 103].

#### miRNA Interactions with TrkA, TrkB, TrkC, and P75NTR

Several miRNAs regulate the TrkA, TrkB, TrkC, and P75NTR signaling pathways [104]. Alterations in the NGF/TrkA signaling pathway are important in neuroblastoma cell differentiation and regression. On the other hand, alteration of miR-92a expression levels is related to the biological behavior of neuroblastoma cells. Higher miR-92a expression values increase the proliferation and migration of human neuroblastoma cells via downregulation of TrkA [105]. The impact of miRNAs on TrkB was evaluated using SHSY5Y cells. miR-216b regulates TrkB-Shc through binding to 3’ UTR [104]. A study on early brain injury after subarachnoid hemorrhage indicated that the administration of human umbilical mesenchymal stem cells-derived miR-206-knockdown exosomes impedes brain injury via the modulation of the BDNF/TrkB/CREB signaling pathway [106].

The results of the human genetic analysis revealed that the expression of miR-185 may impact neurodevelopment through the regulation of the NTR3 gene [47, 107]. Furthermore, CNS damage can target p75NTR and lead to neuronal apoptosis and cell death. Reduction in miR-592, a key regulator of p75NTR, modulates neuronal injury and reduces cell apoptosis after ischemic insults [108]. Furthermore, miR-18a downregulates TrkA and p75NTR mRNA levels in neuroblastoma cell culture [109]. Experimental studies have shown that miR-141 binds to NGF receptor-associated protein 1 mRNA and suppresses the NGF/p75NTR signaling (Table 2) [110].

### Effect of NTs on miRNA Expression

NTs regulate the expression of miRNAs through the activation of various specific TFs, such as NF-kB and CREB. Activation of Trk receptors leads to upregulation of the ERK/CREB signaling pathway, which is involved in the regulation of primiR-212/132 transcription [12]. Besides, the activation of p75NTR can lead to the activation of the NF-kB pathway [111]. The activation of the ERK1/2 and CREB signaling pathways implicated in the NGF-induced expression of miRNAs can promote NGF-related cell survival. For instance, NGF induces miR-221/222 expression through the activation of the ERK1/2 pathway and results in a reduction of pro-apoptosis protein and cell survival (Tables 3 and 4) [112].

**Table 3:** Effects of neurotrophins on microRNAs expression

Neurotrophin	*miRNA*		Types of Research	Types of cell or model	Ref
	Upregulation	*Downregulation*			
NGF	*miR-221/222*	-	In vitro	PC12 cell line	112
	miR-34a	-	In vitro	PC12 cell line	54
	miR-34, miR-181a, miR-200, miR-326	miR-106b, miR-126, miR-139-3p, miR-143, miR-210, miR-532-3p	In vitro	PC12 cell line	59
	-	miR-541	In vitro	PC12 cell line	114
	miR-132	miR-221	In vivo	Sprague–Dawley rats	116
	-	miR-494	In vitro	Human corneal epithelial cell	117
	-	Let-7b, Let-7d, Let-7i, miR-98	In vitro	Muller cells isolated from retina	120
	-	miR-21	In vitro	PC12 cell line	121
	-	miR-181d	In vitro	Primary sensory neurons	119
BDNF	miR-212	-	In vitro	Cortical neuron culture	12
	miR-132	-	In vitro	Cortical neuron cell culture	126
	miR-125b	-	In vitro	SH-SY5Y cells	125
	-	miR-134	In vitro	Primary hippocampal cultures	130
	-	miR-155-5p	In vivo	Experimental autoimmune encephalomyelitis mice model	153

**Table 4:** miRNAs that show altered expression following NGF treatment of cells. *NGF*, nerve growth factor

miRNAs alteration	Types	Ref
miRNA upregulation	miR-34a, miR-34, miR-181a, miR-200, miR-326, *miR-221/222*	54, 59, 112
miRNA downregulation	miR-106b, miR-126, miR-139-3p, miR-143, miR-210, miR-532-3p, miR-541, miR-494, miR-181d, miR-98, Let-7b, Let-7d, Let-7i, miR-21	59, 114, 117, 119, 120, 121

In PC12 cells, sustained mitogen-activated protein kinase/ERK activity and activation of transcription factor activator protein 1 in response to NGF, positively regulate miR-21, which plays an important role in brain development. miR-21 also contributes to the activation of the NGF signaling [113]. Treatment of PC12 cells with NGF modulates the expression of miR-29c, miR-93, miR-212/132, miR-103, miR-30c, miR-691, miR-207, and miR-709 [60]. NGF causes an increase of synapsin I expression via downregulation of miR-541, which plays a crucial role in neurite outgrowth and particularly localizes in axon membrane (Tables 3 and 4) [114]. The proliferation and terminal differentiation of neuronal cells are regulated by the NGF receptor, TrkA, as well as by downstream signaling cascades, including Ras–MAPK, PI3K–Akt pathways, and inositol triphosphate-mediated calcium release [115]. An in vivo study on hypersensitive bladder demonstrates that increased NGF expression is associated with upregulation and downregulation of miR-132 and miR-221, respectively (Table 3) [116]. BDNF is the main regulator of neuron survival with an inhibitory effect on neuronal apoptosis through the activation of the PI3K/Akt pathway. It has been suggested that dysregulation of BDNF-miRNA interaction could result in apoptosis [90].

#### NT Receptor Signaling Interaction with miRNAs

NGF is one of the key modulators of miRNA [59, 60]. The PC12 cell line has been used as a model for the study of the interaction between NGF and miRNAs expressions. Treatment of PC12 cells with NGF upregulates the expressions of miR-34, miR-181a, miR-200, and miR-326 and downregulates the expressions of miR-106b, miR-126, miR-139-3p, miR-143, miR-210, and miR-532-3p (Table 3, 4) [59]. An in vitro study on the role of NGF in the proliferation of human corneal cells revealed that NGF downregulates miR-494 and thereby restores its direct target, Cyclin D, a protein required for the progression of the G1 phase of the cell cycle (Tables 3 and 4) [117]. NGF also downregulates miR-23b by a TF named c-Myc [118]. In primary sensory neurons, NGF inhibits miR-181d-mediated suppression of microtubule-associated protein 1B and calmodulin and consequently leads to axonal elongation (Table 3 and 4) [119]. Differentiation of NGF-treated Muller cells toward neurons is associated with the inhibition of miR-98 as well as Let-7b, Let-7d, and Let-7i (Tables 3 and 4) [120]. Moreover, NGF induces miR-34a expression via the inhibition of tumor suppressor P53 and maintains mature neural cells in the G1 phase (Table 3, 4) [54]. NGF deprivation suppresses miR-21 levels which consequently leads to the elevation of cell division cycle 25 homolog A, caspase activation, and neural death (Tables 3 and 4) [121].

miRNAs can be also regulated by BDNF. It has been reported that miR-1 is dysregulated after BDNF gene deletion in neurons of the dorsal root ganglion [122]. Furthermore, BDNF increases the ratio of miR-212/132 in cortical neuron culture. These miRNAs are regulated by the ERK pathway and particularly downstream effectors mitogen and stress-activated kinase1 and CREB (Tables 3 and 5) [12]. Several studies have found that increased expression of miR-29 controls the upregulation of miR-145 following BDNF-induced SH-SY5Y cell differentiation [123]. miR-134 inhibits LIM kinase-1 (LIMK1) which is crucial for the size of a dendritic spine. BDNF prevents the inhibitory effect of miR-134 on LIMK1 expression and maintains synaptic plasticity [124]. miR-125b is involved in neuroblastoma cell differentiation. RA and BDNF promote miR-125b expression and increase neurite outgrowth (Tables 3 and 5) [125]. In cortical neuron cell culture, BDNF upregulates miR-132 expression via the MAPK/ERK1/2 pathway and leads to increased neurite growth. Interestingly, downregulation of the MAPK/ERK pathway inhibits the BDNF-dependent increase of miR-132. The increase of miR-132 expression through MAPK/ERK1/2 is essential for BDNF-dependent overexpression of postsynaptic proteins, particularly N-methyl-D-aspartate 2A and glutamate receptor 1 (Tables 3 and 5) [12, 126].

**Table 5:** miRNAs that show altered expression following BDNF treatment of cells. *BDNF*, brain-derived neurotrophic factor

miRNAs alteration	Types	References
miRNA upregulation	miR-212, miR-124, miR-132, miR-125b	12,162, 126, 125
miRNA downregulation	miR-134	130

The interaction between BDNF and miR-140 plays a key role in astrocyte proliferation following an injury to the spinal cord [127]. Furthermore, regulatory interaction between BDNF and miRNAs could modulate the proliferation of cancer cells. After treatment with cisplatin, a higher miR-16 expression associated with greater BDNF levels significantly reduces cancer cell differentiation and growth [128]. Radiotherapy on abdomen malignancies by affecting gut flora results in cognitive impairments. miR-34a-5p upregulation in the small intestine and peripheral blood leads to BDNF reduction in the hippocampus and subsequently cognitive dysfunction. Intravenous injection of miR-34a-5p antagomir can prevent gut flora changes and cognitive abnormalities (Table 2) [129]. Moreover, BDNF improves cell survival and inhibits apoptosis in hypoxic-hypoglycemic hippocampal neurons through the activation of TrkB and the inhibition of miR-134 expression. This BDNF effect could be mediated through the modulation of the TrkB/miR-134 pathway (Tables 3 and 5) [130].

## The Role of NTs-miRNA Interaction in Neuropsychological Disorders

NTs-miRNAs interplay plays a key role in the modulation of neural regeneration as well as in cognitive functions [131, 132]. Various studies have suggested that circulating miRNAs can serve as early diagnosis and prognostic biomarkers in neurodegenerative and neuropsychiatric diseases [45, 133]. The mRNA levels of BDNF, NT4, and specific miRNA in peripheral blood mononuclear cells (PBMC) could potentially serve as biomarkers of CNS inflammation and neurodegenerative processes [134]. In the following, we will discuss the role of NTs-miRNA interactions in different neuropsychiatric disorders.

### Role of NTs-miRNA Interaction in Various Neurological Diseases

#### Epilepsy

**Fig. 1:**
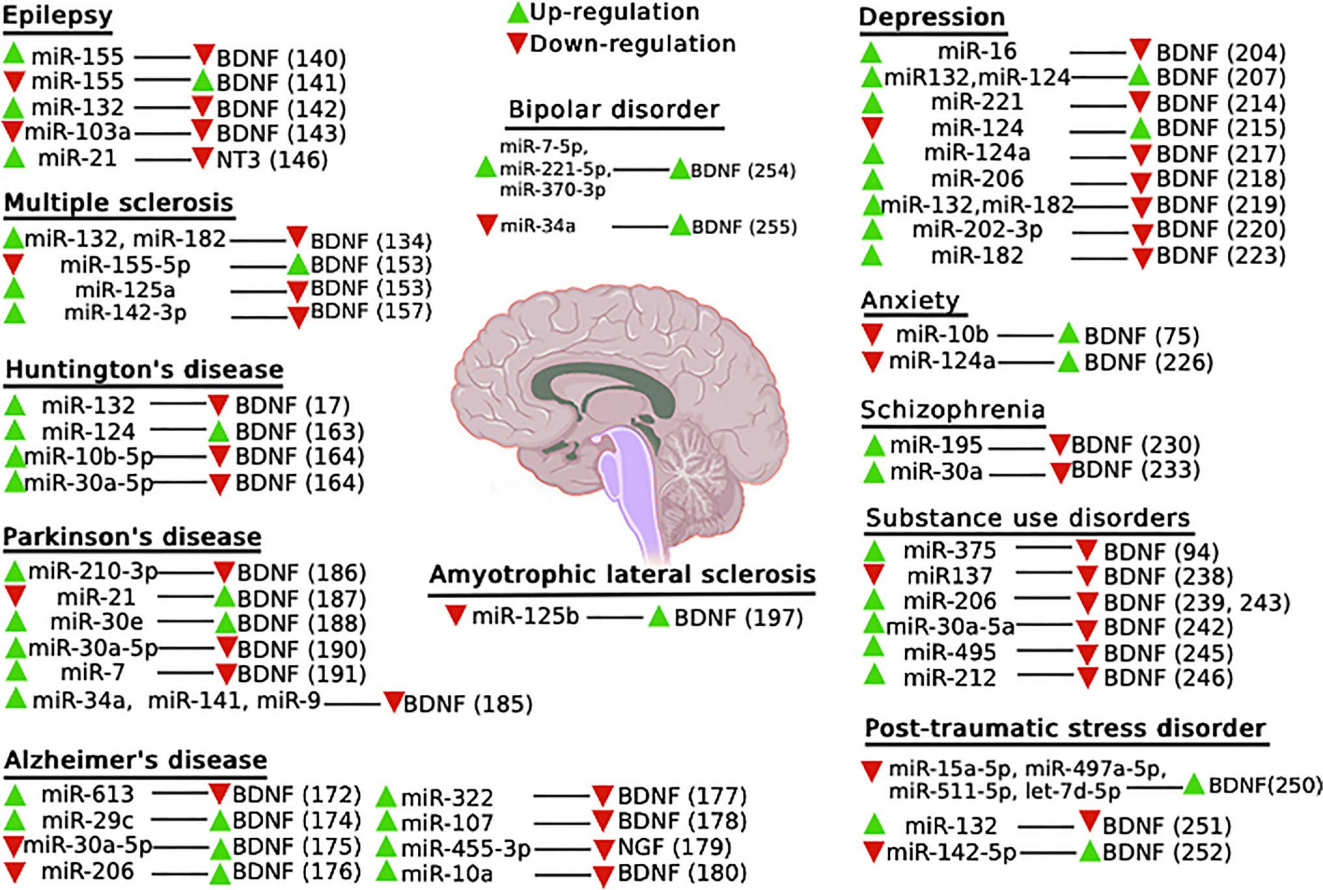
The interaction between various microRNAs and neurotrophins in different neuropsychological disorders

Dysregulation of NTs-miRNAs interaction is involved in the pathogenesis of epilepsy [135]. Targeting NTs-miRNAs interaction affects several biological processes and could be a strategy for efficient intervention following a potential epileptogenic insult [135, 136]. The BDNF/TrkB signaling plays a modulatory role in the brain’s dynamic state leading to greater excitability of mesial temporal lobe epilepsy (MTLE) [137, 138]. An enhancement of the BDNF/TrkB signaling, mediated via BDNF overexpression in the hippocampus, contributes to epileptogenesis in MTLE [139]. Moreover, overexpression of miR-155 significantly suppressed the BDNF and TrkB protein expression and exhibited a neuroprotective effect on epilepsy-induced neuronal damage via the PI3K/Akt/mammalian target of rapamycin (mTOR) signaling pathway in patients with MTLE (Fig. 1) [140]. Application of miR-155 antagonist significantly enhanced the expression of BDNF both at mRNA and protein levels in an animal MTLE model and resulted in the reduction of epileptiform burst discharges and seizure-like behaviors (Table 2, Fig. 1) [141]. Furthermore, upregulation of miR-132 promotes epileptogenesis via the BDNF-TrkB signaling in the primary cultures of hippocampal neurons (Fig. 1) [142]. Inhibition of miR-103a suppresses astrocyte activation in the hippocampus and improves neuronal injury by downregulation of the BDNF gene (Table 2, Fig. 1) [143]. miR-21 expression in the hippocampus is significantly increased after seizures. Enhancement of miR-21 is associated with a decrease in the inhibitory effect of NT3 and an increase in neuronal apoptosis. A significant enhancement of the expression of passenger strand miR-21 compared to mature miR-21 has been observed in the rat hippocampus after pilocarpine-induced status epilepticus. An inverse relationship has been observed between miR-21 and NT3 mRNA levels in hippocampal neurons after status epileptic. Targeting of NT3 by mature miR-21 could potentially result in a greater transforming growth factor-beta receptor expression and contributes to epileptogenesis [144–146]. A higher expression of miR-21 could downregulate the expression of NT3 (Table 2, Fig. 1) [143, 147]. Therefore, miR-21 has been suggested as an ideal target for the modulation of the NT3 signaling in the hippocampus following status epilepticus [146].

#### Multiple Sclerosis

The dysregulation of NTs-miRNAs interaction may influence the inflammatory process in multiple sclerosis (MS) [148]. However, limited investigations were designed to investigate the interaction between NTs and miRNAs in MS [149]. It has been indicated that different NTs, such as BDNF, might produce within active MS lesions [150]. BDNF receptors have been found in reactive astrocytes and neurons in active MS plaques [151]. NGF can improve axon regeneration, synaptogenesis, cell survival, oligodendrocyte differentiation, and oligodendrocyte precursor proliferation in the sclerosis plaques. NGF also promotes the production of BDNF and regulates key proteins essential for myelination [152]. In the experimental autoimmune encephalomyelitis model, it has been shown that BDNF mRNA correlated negatively with pro-inflammatory miR-155-5p expression levels (Fig. 1) [153]. Studies conducted on PBMCs obtained from patients with MS have revealed a decrease in BDNF mRNA expression and simultaneous increases in miR-132 and miR-182-5p values (Fig. 1) [134]. Moreover, the expression of miR-125a, miR-146b, and miR-200c significantly increased, while the expression of miR-328, miR-199a, and miR-152 markedly decreased in peripheral blood of patients with MS [135, 151]. BDNF mRNA was identified as a target of miR-99b and miR-125a. Interestingly, an inverse association was reported between miR-125a and BDNF in an experimental MS model (Fig. 1) [153]. In MS lesions, autoimmune and mesenchymal stem cells protect specific cell populations and suppress the formation of new lesions through the release of NGF and GDNF at the lesion sites [154]. Various types of microglia play complex roles in neuroinflammation and regeneration processes in MS [155]. miR-142-3p is one of the highly upregulated miRNAs in microglia in response to various pathological insults, such as the inflammatory process in MS [156]. miR-142-3p modulates BDNF expression via its target calcium/calmodulin-dependent kinase 2a and modulates the expression of proinflammatory mediators (Fig. 1) [157].

#### HD

The underlying molecular mechanisms of HD, such as alterations in synaptic plasticity, gene expression, neurotransmitter signaling, NTs, and miRNAs, can be considered potential therapeutic targets [131]. The abnormal expression of global miRNAs or specific miRNAs has been determined in different regions of the brains of HD-affected subjects [131, 158]. The striatum has been identified as a primary site of degeneration in HD. It has been hypothesized that altered BDNF delivery from the neocortex to the striatum plays a role in the pathophysiology of HD [159]. The decreased BDNF release and transport in neocortical neurons lead to insufficient trophic support of the striatum and enhance the vulnerability of striatal neurons and synapses in a knock-in mouse model of HD [160, 161].

A limited number of studies have investigated the effect of NTs-miRNA interaction in HD [131]. The modulatory interplay between miR-132, BDNF, and MeCP2 plays a key role in the pathophysiology of HD. miR-124 is positively regulated by BDNF in HD (Table 5) [162]. Furthermore, there is regulatory feedback between miR-132 and MeCP2, as well as with its downstream target BDNF in HD. Upregulation of miR-132 leads to the suppression of MeCP2 and BDNF transcript levels and consequently striatal cell death (Fig. 1) [17]. Furthermore, miRNA-124 may contribute to neurogenesis by regulating NTs in HD. miR-124 increases the value of BDNF, promotes neurogenesis, and improves neuronal survival in the striatum in an animal model of HD (Table 2, Fig. 1) [163]. Studies on the miRNA profile of patients with HD revealed upregulation of miR-10b-5p and miR-30a-5p, which leads to downregulation of BDNF and neuronal death. CREB1 is the predicted target gene of these two miRNAs in HD. miR-10b-5p has a neuroprotective effect in response to the mutation in HD (Fig. 1) [164]. Since HD is an inherited neurodegenerative condition through different mechanisms, such as abnormality and misfolding of proteins, mitochondrial dysfunctions, and degradation of misfolded protein, targeting NTs-miRNA interaction may provide a potential treatment option for HD [165, 166]. Moreover, it has been shown that the p65 subunit of NF-κB regulates miR-146a in an HD experimental model [167].

#### AD

miRNAs play an important regulatory role in different neurodegenerative diseases [36]. BDNF is crucial to the maintenance of neocortical network activities and its dysfunction contributes to memory impairment in AD [168]. NGF also plays a key role in the maintenance of neural structural integrity and function and enhances cell survival and regeneration in subjects with age-related diseases, such as AD [169]. The epigenetic mechanisms, like DNA methylation and miRNA alterations, can regulate the expression of NTs in patients with AD [131]. An elevated value of different miRNAs in human prefrontal neocortical tissue was associated with a reduced value of BDNF [170].

miRNAs are gene modulatory molecules with neuroprotective roles in the development of AD. The levels of different miRNAs are associated with the expression of various AD-related proteins [171]. Analysis of CSF and serum of patients with mild cognitive impairment and dementia of AD type, as well as the hippocampus of an AD mice model, indicated that miR-613 downregulates the expression of BDNF through directly targeting 3′ UTR (Table 2, 6, Fig. 1) [172]. Lower values of BDNF have been identified in the neocortex and hippocampus in AD [173]. CSF analysis of patients with AD indicated that the expression of miR-29c was positively associated with the protein expression of BDNF; suggesting its effect on neuronal proliferation through the regulation of BDNF expression (Fig. 1) [174]. Neuropeptide Y, a potent orexigenic neuromodulator in the brain, increased BDNF mRNA and protein expression by inhibiting miR-30a-5p in an in vitro model of AD (Fig. 1) [173, 175]. Increased miR-206 brain level has been observed in the mouse model of AD, whereas its reduction promoted the BDNF levels and improved cognitive functions (Fig. 1) [176]. miR-322 produces tau phosphorylation by negatively regulating BDNF-TrkB signal activation in AD (Fig. 1) [177]. Moreover, intraventricular application of amyloid-β1-42 (Aβ1-42) reduced the miR-107 level in mice. However, the administration of miR-107 mimic prevented the impairments of spatial memory and synaptic plasticity as well as the cell loss caused by Aβ neurotoxicity through the inhibition of the BDNF-TrkB signaling pathway (Fig. 1) [178]. An enhancement of miR-455-3p expression has been reported in patients with AD, which was associated with Aβ pathologies and modulation of NGF (Fig. 1) [179]. Moreover, miR-10a overexpression inhibits hippocampal synapse remodeling and cell proliferation and promotes apoptosis in AD rats through the inhibition of the BDNF-TrkB signaling pathway (Fig. 1) [180].

#### PD

miRNAs have been reported to implicate in pathways related to the pathophysiology of PD [181]. Previous studies indicated that downregulation of Dicer expression in dopamine neurons may cause dysregulation of various miRNAs linked to PD-associated genes, such as miR-133b, and regulates the function of aged dopaminergic neurons [182]. Furthermore, NTs are involved in multiple signaling cascades that play roles in the progression of PD pathology [183]. The BDNF/TrkB signaling is essential for the survival and maturation of the nigrostriatal dopaminergic neurons. The BDNF inhibits neuronal apoptosis and promotes the maturation of functional dopaminergic neurons [131, 184]. Upregulation of miR-34a, miR-141, and miR-9 is associated with downregulation of Sirt1, B-cell lymphoma protein 2, and BDNF mRNA in an in vitro PD model (Fig. 1). Importantly, this study has shown that miR-34a could become the target of the alteration of human BDNF levels for the treatment of PD (Table 2) [185].

In the PD neuron models, upregulation of miR-210-3p reduces BDNF production and results in neuronal damage (Table 2, Fig. 1) [186]. Elevated miR-21 levels and reduced peroxisome proliferator-activated receptor alpha (PPARα) values have been observed in patients with PD. A combined application of an omega-3 fatty acid and aspirin effectively promoted the expression of PPARα protein as well as BDNF and GDNF protein via the inhibition of miR-21 in SH-Y5Y cells (Fig. 1) [187]. miR-30e has significantly downregulated in the substantia nigra in a mouse model of PD. Application of the miR-30e agomir restored the sustained decreased BDNF production in these mice, which was associated with improved motor behavioral function and neural network activity (Fig. 1) [188]. Furthermore, the link between BDNF and various miRNAs, such as miR-210-3p, miR-34a, miR-141, miR-9, miR-21, and miR-30, in PD pathology, has been suggested [189]. A potential role of dysregulation of hypothalamic BDNF and miR-30e via the modulation of the melanocortin-4 receptor in the pathophysiology of PD has been suggested [131]. Moreover, miR-30a-5p reduces BDNF values and exerts a neurotoxic role on dopaminergic neurons in PD (Fig. 1) [190]. miR-7 also regulates the expression of BDNF through an autoregulatory mechanism in the early stages of neuronal damage in the atrazine-induced rat model of PD (Fig. 1) [191]. Alterations of miR-134 and miR-141 modulate the expression of mesencephalic astrocyte-derived neurotrophic factor and cerebral dopamine neurotrophic factor that play a role in the pathophysiology of several neurological disorders, including PD [192].

#### Amyotrophic Lateral Sclerosis

Enhancement of BDNF levels has been considered one of the main strategies to stop or prolong the progression of amyotrophic lateral sclerosis (ALS). The modulation of the BDNF/TrkB pathway under certain conditions exerts neuroprotective effects on motor neurons against various pathological insults [193–195], probably via the inhibition of apoptosis and restoring the impaired calcium homeostasis [196]. Dysregulation of several miRNAs, such as miR-132, miR-125b, miR-34a, and miR-504, has been determined in patients with ALS [197–199]. Microglia are a possible source of dysregulated miRNAs in ALS [200]. The dysfunction of microglial downregulates BDNF/TrkB signaling in motor neurons of ALS mice [201]. The inhibition of miR-125b exerts a neuroprotective effect on motor neurons via both reduction of pro-inflammatory mediators and the stimulation of microglia activators, such as BDNF, in ALS (Fig. 1) [197]. Different miRNAs, such as miR-320a, miR-424-5p, and miR-503, modulate the differentiation of mesenchymal stromal cells induced to express high levels of neurotrophic factors and potentially could be used as a biomarker in ALS clinical trials [202].

### Role of miRNAs and NTs in Psychological Disorders

Based on the function of NTs in neuronal development and synaptic plasticity, a growing body of relevant evidence suggests the implication of NTs in the pathophysiology of various psychological disorders [203]. Furthermore, several studies have suggested that alterations of miRNAs expression profiles could contribute to the pathophysiology of psychological disorders, such as schizophrenia, depression, anxiety, drug abuse, PTSD, and bipolar disorder [204–206]. Besides, some studies indicate the importance of the NTs-miRNAs interactions in the development and progression of several neuropsychiatric disorders [207].

#### Depression

Alterations in the expression of different NTs contribute to the pathophysiology of depression [208, 209]. It has been suggested that the enhancement of the NTs signaling has a strong potential for the treatment of depression and the molecules-derived NTs pathways might be considered a biomarker for depression [210, 211]. BDNF exerts region-dependent antidepressant effects. Shati/Nat8l, an N-acetyltransferase in the dorsal striatum, can regulate BDNF via epigenetic regulations. The targeting of the Shati/Nat8l-BDNF pathway could be a potential therapeutic target for the treatment of depression [208]. Furthermore, it has been found that the level of NGF mRNA in the brain is correlated with anxiety and depression symptoms [212]. Evidence from experimental investigations and postmortem studies suggests that alterations of miRNAs contribute to the pathology of depression. Both upregulation and downregulation of several miRNAs have been reported in patients with depression [213]. miR-221 involves in the development of depression. It targets the wingless-type MMTV integration site family member 2, which results in the decreased activity of the CREB/BDNF signaling pathway in the hippocampus (Table 2, Fig. 1) [214]. miR-124 is a type of miRNA abundantly expressed in the hippocampus and directly targets the glucocorticoid receptor expression in the human embryonic kidney-293 cells. Downregulation of miR-124 may provide a strategy for the treatment of depression by activating the BDNF-TrkB, ERK, and CREB signaling pathways in the hippocampus. Under long-time exposure to stressful conditions, glucocorticoid hormones may cause depression via the regulatory effect of miR-124 on BDNF (Table 2, Fig. 1) [215]. In vivo study of corticosterone-induced depressive-like mice indicated that upregulation of miR-124 is required for the inhibition of the CREB-TrkB signaling pathway in the hippocampus [216]. Changes in miR-124a might participate in the induction of depressive-like behavior through direct regulation of BDNF gene expression in stressed rats (Fig. 1) [217]. Moreover, alterations in miR-132 and miR-124 values in non-treated and citalopram-treated patients with depression have shown that enhancement of both miRNAs increases plasma BDNF values (Fig. 1) [207].

Ketamine, a potent anti-depressive substance, decreases miR-206 expression in the hippocampus and miR-206 upregulation significantly reduces the ketamine-dependent increase of BDNF (Fig. 1) [218]. In maternal deprivation-induced depressive-like behaviors, overexpression of miR-16 is accompanied by a significant decrease in BDNF (Fig. 1) [204]. Furthermore, a decrease in BDNF levels was associated with increased values of miR-132 and miR-182 in patients with depression; suggesting a potential role of serum BDNF and its related miRNAs as diagnostic biomarkers (Table 2, Fig. 1) [219]. Upregulation of miR-202-3p significantly increases depressive-like behaviors, decreases the expression of BDNF, and reduces hippocampal damage in rats (Table 2, Fig. 1) [220]. Moreover, interactions between miR-132 and MeCP2 modulate the hippocampal BDNF protein expression in a rat model of chronic stress-induced depression [221]. Furthermore, miR-26a-3p plays a key role in the hippocampal neuronal network alterations in a chronic unpredictable mild stress (CUMS)-induced rat model through its regulatory effects on BDNF and the phosphatase/tensin homolog/PI3K/Akt signaling pathway [222]. In vitro neuronal studies and in vivo models of CUMS revealed that miR-182 directly inhibits BDNF and leads to lower CREB levels and depression-like behaviors (Table 2, Fig. 1) [223].

#### Anxiety Disorders

Experimental and clinical studies indicate the involvement of BDNF in anxiety disorders. Different types of stressors lead to a reduction of BDNF expression values [224]. Furthermore, several studies have reported the association of NTR3 activation with the pathophysiology of anxiety disorders. Consequently, it has been demonstrated that miR-9 and miR-125 regulate the expression of the t-NTR3 isoform in anxiety-like behaviors [225]. It has been reported that the reduction of miR-124a expression in the hippocampal dentate gyrus leads to decreased anxiety-like behavior, which is inversely correlated with the expression of its target gene, BDNF (Fig. 1) [226]. In the chronic unpredictable stress-induced depression rat model, miR-10b downregulation and BDNF upregulation have been shown in the hippocampus (Fig. 1) [75].

#### Schizophrenia

Both experimental and clinical investigations suggest that alterations in NTs and miRNAs in certain brain regions are implicated in the pathophysiology of schizophrenia [227, 228]. miR-137 regulates the expression of schizophrenia-associated genes and contributes to the regulation of neuronal response by targeting the PI3K-Akt-mTOR branch of neuregulin-1/ErbB and BDNF signaling [229]. In the prefrontal cortex of patients with schizophrenia, downregulation of neuropeptide Y and somatostatin mRNA values are associated with increased miR-195 levels and decreased BDNF expression (Fig. 1) [230]. The correlation between miR-195 and BDNF changes may play a role in GABAergic neurotransmission abnormalities and influence cognitive impairments of patients with schizophrenia [230, 231]. Furthermore, the alterations of the miR-30a family in the prefrontal cortex of patients with schizophrenia are associated with changes in BDNF levels (Fig. 1) [170, 232, 233]. Early growth response 3 (EGR3) and some miRNAs play a modulatory role in the schizophrenia regulatory neuronal network [234]. EGR3, a downstream gene of different signaling pathways, is triggered by various NTs, such as NGF and BDNF [235].

#### Substance Use Disorders

Dysregulation of various miRNAs-NTs interactions is implicated in the pathophysiology of drug abuse. There are growing studies that explore the interplay between drug abuse and NTs biological action in various brain regions. The expression of striatal BDNF correlates with the expression of CREB, TrkB, and pri-miR-132 following amphetamine application in rats [236]. Heavy alcohol use causes upregulation of some miRNAs and consequently regulates BDNF values [237]. Adolescent intermittent ethanol exposure increased the miR-137 expression level in the amygdala. Application of miR-137 antagomir in the amygdala decreased BDNF levels and improved anxiety-like behavior following alcohol consumption in rats (Fig. 1) [238]. Ethanol exposure decreased BDNF and enhanced miR-206 expression in different mice brain structures, including the medial prefrontal cortex, central amygdala, and hippocampus (Fig. 1) [239]. Induction of miR-206 expression and its modulation of BDNF after prolonged brain exposure to ethanol could alter the synaptic plasticity implicated in the cognitive control of alcohol consumption and lead to alcohol dependence [240]. In an investigation of brain tissue of alcohol-dependent rats, a significant alteration of miR-101b and BDNF expression has been reported [241]. Overexpression of miR-30a-5p in association with reduced levels of BDNF in the medial prefrontal cortex can play an important role in the transition from moderate to excessive alcohol intake (Fig. 1) [242]. Furthermore, ketamine-induced neural death and toxicity are accompanied by miR-375 upregulation that directly downregulates BDNF expression in hESC (Fig. 1) [94]. miR-206 downregulates BDNF levels in both neuronal cell culture in vitro and the hippocampus in vivo (Fig. 1) [243]. The interaction between miR-206 and BDNF expression in the nucleus accumbens has been suggested to control the reconsolidation of cocaine-associated memory [244]. Overexpression of miR-495 and its direct effect on BDNF value in the nucleus accumbens has been observed after acute cocaine administration in mice (Fig. 1) [245]. Alterations of miR-212 may regulate cocaine intake through the modulation of striatal CREB and MeCP2 signaling; which consequently decreases BDNF protein levels and decrease the motivational effects of cocaine (Fig. 1) [246–248]. Moreover, changes in miR-124, miR-181a, and let-7d as well as BNDF values in the mesolimbic dopaminergic system are implicated in a complex feedback loop with cocaine-induced plasticity [249].

#### PTSD

In a mouse model of PTSD, a strong reduction of miR-15a-5p, miR-497a-5p, miR-511-5p, and let-7d-5p levels in the medial prefrontal cortex were correlated with two key PTSD-related genes, FKBP5 and BDNF (Fig. 1) [250]. In another study on a rat model, it was suggested that miR-132 is involved in PTSD and led to the reduction of BDNF expression through MeCP2. The application of its antagomir can improve anxiety behavior and upregulates MeCP2 and BDNF (Fig. 1) [251]. Furthermore, rats exposed to stress exhibited enhanced miR-142-5p values in the amygdala, which was accompanied by a reduction in levels of Npas4, an activity-regulated transcription factor. The inhibition of miR-142-5p in these rats reduced anxiety-like behaviors and enhanced Npas4 and BDNF expressions (Fig. 1) [252].

#### Bipolar Disorder

An investigation of 288 patients with bipolar disorder revealed that interaction between miR-206 and BDNF polymorphism increases the risk for bipolar disorder and treatment response to various drugs [253]. Furthermore, it has been shown that the expression of peripheral miR-7-5p, miR-221-5p, and miR-370-3p are correlated to BDNF levels in 98 patients with bipolar patients (Fig. 1) [254]. Lithium is the major medication for mood stabilizing in bipolar disorder, which exhibits neuroprotective effects. Experiments on lithium pretreated SH-SY5Y human neuroblastoma cells provide evidence that lithium significantly decreases the expression of miR-34a, which is correlated with BDNF and anti-apoptotic protein BCL2 levels (Fig. 1) [255].

## Conclusion

A series of experimental and clinical studies yields promising results suggesting the pivotal roles of NTs and miRNAs interactions in the pathophysiology of various neuropsychological disorders. Understanding how NTs and miRNAs interactions modulate pathological processes in different brain disorders helps design novel diagnostic and therapeutic approaches. Modulation of several brain-specific miRNAs could alter the expression and function of different NTs and vice versa. Changes in NTs and miRNAs expression and function contribute to brain hyperexcitability in epileptic patients, neurodegenerative processes in patients with AD, PD, HD, or ALS, demyelination in patients with MS, and perturbation in neural circuits and neurotransmitters in patients with different psychological disorders. On the other hand, alterations in NTs and miRNAs interactions could regulate neuronal and synaptic hyperexcitability, exert neuroprotective effects, promote myelination, and improve cognitive and behavioral impairment. Future studies are required to discover the exact mechanism of interplay between NTs and miRNAs in physiological and pathological conditions.

## Data Availability

Not applicable.

## Abbreviations

AD: Alzheimer’s disease
ALS: Amyotrophic lateral sclerosis
BDNF: Brain-derived neurotrophic factor
CREB: CAMP response element-binding protein B
CNS: Central nervous system
CSF: Cerebrospinal fluid
CUMS: Chronic unpredictable mild stress
EGR3: Early growth response 3
ERK1/2: Extracellular-signal-regulated kinase 1/2
hESC: Human embryonic stem cell
HD: Huntington’s disease
LIMK1: LIM kinase-1
mTOR: Mammalian target of rapamycin
MeCP2: Methyl CpG binding protein 2
miRNAs: MicroRNAs
Ras/Raf/MAPK: Mitogen-activated protein kinase
MS: Multiple sclerosis
NGF: Nerve growth factor
NTs: Neurotrophins
NT3: Neurotrophin-3
NT4: Neurotrophin-4
NTR3: Neurotrophic receptor tyrosine kinase 3
NF-κB: Nuclear factor kappa B
PD: Parkinson’s disease
PC12: Pheochromocytoma cell line
PI3K-Akt: Phosphatidylinositol 3-kinase-protein kinase B
PBMCs: Peripheral blood mononuclear cells
PPARα: Peroxisome proliferator-activated receptor alpha
PTSD: Post-traumatic stress disorder
p75NTR: P75 neurotrophin receptor
RA: Retinoic acid
Sirt1: Silent information regulator 1
TF: Transcription factor
TFs: Transcription factors
Trk: Tropomyosin-related kinase
t-NTR3: Truncated isoform of NTR3
3′ UTR: 3′ Untranslated region
